# ϕSa3mw Prophage as a Molecular Regulatory Switch of Staphylococcus aureus β-Toxin Production

**DOI:** 10.1128/JB.00766-18

**Published:** 2019-06-21

**Authors:** Phuong M. Tran, Michael Feiss, Kyle J. Kinney, Wilmara Salgado-Pabón

**Affiliations:** a Department of Microbiology and Immunology, Roy J. and Lucille A. Carver College of Medicine, University of Iowa, Iowa City, Iowa, USA; Michigan State University

**Keywords:** PR-switch, Sa3int, *Staphylococcus aureus*, beta toxin, biofilm, infective endocarditis, oxidative stress, phage regulatory switch

## Abstract

Phage regulatory switches (phage-RSs) are a newly described form of active lysogeny where prophages function as regulatory mechanisms for expression of chromosomal bacterial genes. In Staphylococcus aureus, ϕSa3int is a widely distributed family of prophages that integrate into the β-toxin structural gene *hlb*, effectively inactivating it. However, β-toxin-producing strains often arise during infections and are more virulent in experimental infective endocarditis and pneumonia infections. We present evidence that in S. aureus MW2, ϕSa3mw excision is temporally and differentially responsive to growth conditions relevant to S. aureus pathogenesis. PCR analyses of ϕSa3mw (integrated and excised) and of intact *hlb* showed that ϕSa3mw preferentially excises in response to hydrogen peroxide-induced oxidative stress and during biofilm growth. ϕSa3mw remains as a prophage when in contact with human aortic endothelial cells in culture. A criterion for a prophage to be considered a phage-RS is the inability to lyse host cells. MW2 grown under phage-inducing conditions did not release infectious phage particles by plaque assay or transmission electron microscopy, indicating that ϕSa3mw does not carry out a productive lytic cycle. These studies highlight a dynamic, and perhaps more sophisticated, S. aureus-prophage interaction where ϕSa3int prophages provide a novel regulatory mechanism for the conditional expression of virulence factors.

**IMPORTANCE** β-Toxin is a sphingomyelinase hemolysin that significantly contributes to Staphylococcus aureus pathogenesis. In most S. aureus isolates the prophage ϕSa3int inserts into the β-toxin gene *hlb*, inactivating it, but human and experimental infections give rise to β-toxin-producing variants. However, it remained to be established whether ϕSa3mw excises in response to specific environmental cues, restoring the β-toxin gene sequence. This is not only of fundamental interest but also critical when designing intervention strategies and therapeutics. We provide evidence that ϕSa3mw actively excises, allowing the conditional expression of β-toxin. ϕSa3int prophages may play a novel and largely uncharacterized role in S. aureus pathogenesis as molecular regulatory switches that promote bacterial fitness and adaptation to the challenges presented by the mammalian host.

## Abstract (precis)

β-Toxin is a sphingomyelinase hemolysin that significantly contributes to Staphylococcus aureus pathogenesis. In most S. aureus isolates the prophage ϕSa3int inserts into the β-toxin gene *hlb*, inactivating it, but human and experimental infections give rise to β-toxin-producing variants. However, it remained to be established whether ϕSa3mw excises in response to specific environmental cues, restoring the β-toxin gene sequence. This is not only of fundamental interest but also critical when designing intervention strategies and therapeutics. We provide evidence that ϕSa3mw actively excises, allowing the conditional expression of β-toxin. ϕSa3int prophages may play a novel and largely uncharacterized role in S. aureus pathogenesis as molecular regulatory switches that promote bacterial fitness and adaptation to the challenges presented by the mammalian host.

## INTRODUCTION

Staphylococcus aureus causes a wide array of diseases, ranging in severity from localized skin and soft-tissue infections to life-threatening diseases like bacteremia, toxic shock syndrome, and infective endocarditis (1). It is also the leading cause of health care-associated infections (2, 3). The ability of S. aureus to facilitate these different infections is due to its wide arsenal of secreted and cell-associated virulence factors (4). Several virulence factors are found within mobile genetic elements such as the staphylococcal chromosomal cassette (SCC), bacteriophages, pathogenicity islands, and plasmids, which accounts for virulence factor variability within staphylococcal strains (5). S. aureus prophages induce lysogenic conversion, and this plays a significant role in S. aureus adaptability and virulence (6–9). The ϕSa3int family of prophages is the most prevalent staphylococcal integrating prophage (10, 11). ϕSa3int prophages encode the immune evasion cluster (IEC) comprised of the immune modulators staphylokinase (SAK), staphylococcal complement inhibitor (SCIN), staphylococcal enterotoxin A (SEA), and chemotaxis inhibitory protein of S. aureus (CHIPS) (11–13). ϕSa3int prophages integrate into the β-toxin-encoding gene *hlb*, resulting in single-conversion (*hlb* interrupted), double-conversion (*hlb* interrupted, SAK^+^), or triple-conversion (*hlb* interrupted, SAK^+^, SEA^+^) events (14–17). Thus, β-toxin was presumed to have little impact on S. aureus pathogenesis.

However, recent evidence indicates that β-toxin promotes host colonization, modulates the immune response to infection, and increases the severity of life-threatening infections like pneumonia and infective endocarditis (11, 18–21). Interestingly, β-toxin-producing variants (β-toxin^+^) arise during or after infection at a higher frequency than under standard laboratory growth conditions (11, 19, 20). β-Toxin^+^ variants are enriched in the sputum of cystic fibrosis patients and in rabbit models in endocarditis vegetations, ischemic livers, and kidney abscesses (20, 22). These studies suggest that β-toxin is preferentially expressed when S. aureus encounters a host. However, there have been no studies that focus on the role of the ϕSa3int prophages as a regulatory mechanism controlling the expression of β-toxin.

Phage-regulatory switches (phage-RSs) are a newly described form of active lysogeny where phage excision functions as a regulatory mechanism for expression of chromosomal bacterial genes without entering the lytic cycle. Several phage-RSs have now been described, including those that activate the competence system master regulator *comK* during Listeria monocytogenes phagosomal escape (23), deactivate the Streptococcus pyogenes mismatch repair system under stress conditions (24), activate late-stage sporulation genes in Bacillus subtilis (25), activate nitrogen fixation in cyanobacteria under nitrogen-limiting conditions (26), promote biofilm formation in Escherichia coli (27), and induce lipopolysaccharide variation in Legionella pneumophila (28). Although incompletely understood, in all these examples prophage excision leads to alteration or regulation of critical bacterial processes.

Here, we tested the concept that ϕSa3int prophages act as novel phage-RSs for the conditional expression of *hlb*. Prophage dynamics and restoration of intact *hlb* was evaluated in the S. aureus methicillin-resistant strain MW2 under *in vitro* growth conditions relevant to S. aureus pathogenesis: bacterial stress *in vitro*, mammalian cell infection, and growth in a biofilm. We provide evidence that ϕSa3mw (the ϕSa3int prophage in MW2) preferentially excises in response to oxidative stress and during biofilm growth but remains as a prophage when in the presence of human aortic endothelial cells. We were not able to detect viable prophage particles by plaque assay or transmission electron microscopy (TEM) under phage-inducing conditions, indicating that ϕSa3mw does not release infectious virions.

## RESULTS

### H_2_O_2_ induces β-toxin production.

S. aureus strains carrying ϕSa3int prophages give rise to bacterial subsets that produce β-toxin at wild-type levels under *in vitro* culture conditions and at higher proportions during infection (20). However, excision dynamics were assessed only by detection of bacteria that were phage cured, and enrichment for β-toxin-producing variants already present in the population was not ruled out. To address this, the temporal dynamics of *hlb* expression (promoter activity) and β-toxin production was assessed during planktonic growth or under bacteriostatic conditions. For this, we generated a β-toxin promoter reporter and a β-toxin translational reporter in the S. aureus strain MW2. The β-toxin promoter reporter strain contains a plasmid carrying the gene for superfolding green fluorescent protein (*sgfp*) under the control of the *hlb* promoter (P*_hlb_-sgfp*). The β-toxin translational reporter strain expresses an *hlb-sgfp* C-terminal translational fusion (β-toxin–sGFP) from its native chromosomal site. In this strain, *hlb-sgfp* can only be transcribed if ϕSa3mw precisely excises to restore the *hlb* sequence. Expression of full-length β-toxin–sGFP was confirmed by Western blotting in overnight cultures of an isogenic nonlysogen strain, MW2ΔϕSa3mw, encoding the translational fusion (see Fig. S1 in the supplemental material).

**FIG 1:**
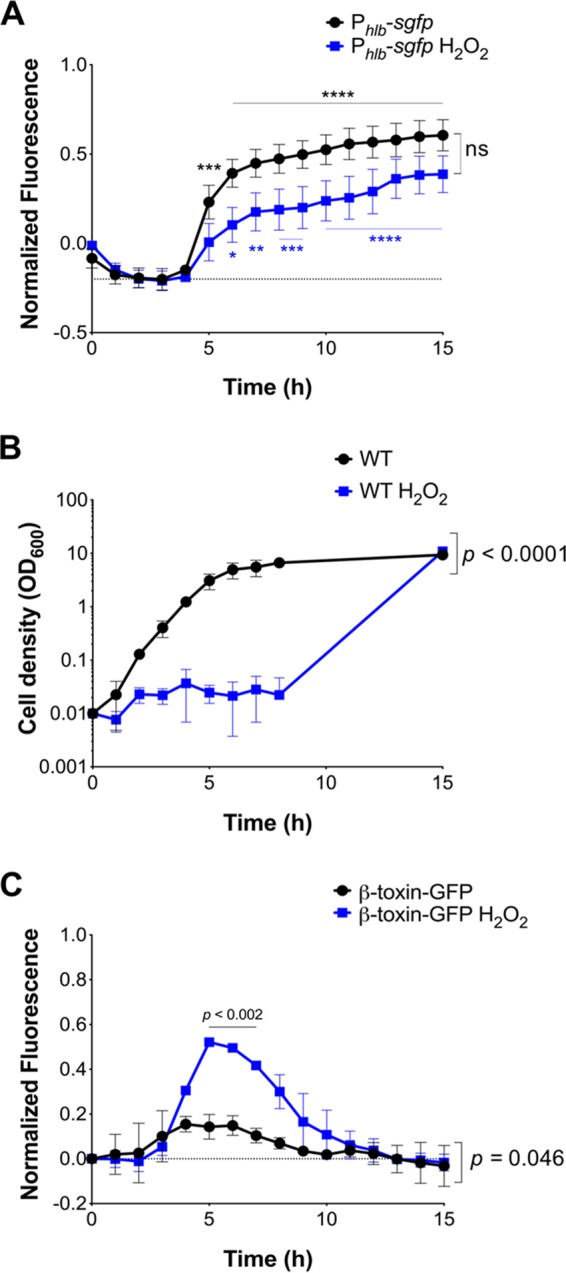
H_2_O_2_ induces β-toxin production under bacteriostatic conditions in a time-dependent manner. S. aureus wild-type (WT) and fluorescent reporter strains were grown in Todd-Hewitt (TH) medium (black) or TH plus 1 mM H_2_O_2_ (blue) overnight. (A) Fluorescence signal over time of S. aureus MW2 ectopically expressing sGFP under the control of the *hlb* promoter (P*_hlb_-sgfp*). (B) Bacterial cell density. (C) Fluorescence signal over time of S. aureus MW2 chromosomally expressing β-toxin fused to sGFP (β-toxin–sGFP). For panels A and C, empty vector control was subtracted from fluorescence readings at corresponding time points to correct for autofluorescence. Results are averages from at least three independent experiments performed in triplicate (means ± standard errors of the means [SEM]). Statistical significance was determined by 2-way ANOVA with Sidak’s multiple-comparison test. (A) TH, *P* = 0.0002 (5 h) (***) and *P* < 0.0001 (6 to 15 h) (****); TH plus H_2_O_2,_ *P* = 0.023 (6 h) (*), *P* = 0.001 (7 h) (***), *P* < 0.0009 (8 to 9 h) (***), and *P* < 0.0001 (10 to 15 h) (****); TH versus TH plus H_2_O_2_ trend lines, not significant (ns); TH and TH plus H_2_O_2_ time effect, *P* < 0.0001. (B) H_2_O_2_ effect on cell density, *P* < 0.0001; time effect, *P* < 0.0001. (C) TH versus TH plus H_2_O_2_ trend lines, *P* = 0.046; TH versus TH plus H_2_O_2_, *P* < 0.0002 (5 to 7 h); TH and TH plus H_2_O_2_ time effect, *P* < 0.0001.

A time course analysis of MW2 containing P*_hlb_-sgfp* shows induction of P*_hlb_* after 5 h of growth in Todd-Hewitt broth (TH) when S. aureus is in late exponential growth phase (Fig. 1A and B). These results are consistent with quorum-sensing regulated gene expression of secreted toxins (29–32) and confirm that S. aureus MW2 possesses a functional *hlb* promoter. Growth of MW2 expressing the β-toxin–sGFP translational fusion also led to significantly higher levels of β-toxin–sGFP during 4 to 8 h of growth, albeit at low levels, indicating that β-toxin production does occur during growth (Fig. 1C and Fig. S2). We then sought to investigate if β-toxin production was differentially induced under bacterial stress. In culture, H_2_O_2_ is bacteriostatic and induces DNA damage by causing double-stranded DNA breaks, a common signal for prophage excision from the host genome (33, 34). As expected, growth of MW2 was halted in the presence of 1 mM H_2_O_2_ for at least 8 h (Fig. 1B). After overnight growth, bacteria recovered, reaching optical cell densities and CFU titers (data not shown) similar to those of cells grown in the absence of H_2_O_2_ (Fig. 1B). During the first 8 h of culture, when S. aureus is under bacteriostatic conditions, *hlb* promoter activity was still detectable over time (Fig. 1A). Importantly, β-toxin–sGFP was detected at significantly higher levels during 5 to 7 h of exposure to H_2_O_2_ than during exponential growth in TH (Fig. 1C). These findings rule out enrichment of phage-cured variants as a sole mechanism for β-toxin production and provide evidence that ϕSa3mw specifically excises in response to bacterial stress.

### ϕSa3mw does not release infective virions after treatment with H_2_O_2_ or mitomycin C.

A criterion for phage-RSs is that their excision does not result in host cell lysis (35). The decrease in detectable β-toxin–sGFP after 8 h of exposure to H_2_O_2_ (Fig. 1C) suggests that ϕSa3mw either reintegrated into the chromosome (stopping β-toxin production) or became lytic, killing the subpopulation producing β-toxin. To address the latter, we first assessed production of infectious ϕSa3mw virions by plaque assay. Supernatants from MW2 either uninduced or induced with H_2_O_2_ (1 mM) or mitomycin C (1 μg ml^−1^) for 4 h were used to infect the indicator strains MW2ΔϕSa3mw and RN4220. RN4220 is a restrictionless laboratory strain susceptible to phage infection. No plaques were visible from any supernatant (data not shown). To confirm these results, we utilized transmission electron microscopy (TEM) to visualize phage particles present in H_2_O_2_- or mitomycin C-treated culture supernatants from MW2 and MW2ΔϕSa3mw (used as a ϕSa3mw-specific control). To ensure that phages were appropriately isolated, ϕ11, a lytic phage propagated in RN4220, was used as a technical control. Both ϕ11 and ϕSa3mw belong to the *Siphoviridae* family of phages (9). While ϕ11 was present at high titers in the processed supernatant, phage particles were not found in any of the conditions tested from MW2 or MW2ΔϕSa3mw (data not shown). Furthermore, MW2 or MW2ΔϕSa3mw treatment with mitomycin C did not result in clearing of the culture, typical of lytic phages (data not shown). Altogether, these findings are consistent with induction of β-toxin production under the conditions tested by a mechanism involving active lysogeny.

### ϕSa3mw temporally excises during planktonic growth and under bacterial stress.

**FIG 2:**
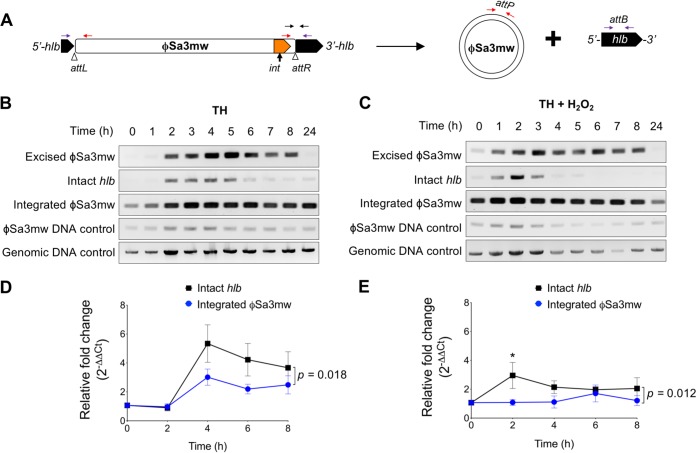
ϕSa3mw differentially excises in the presence of H_2_O_2_. (A) Schematic of ϕSa3mw integrated or excised from *hlb* with primer locations (colored arrows). *attL* and *attR* are the 5′- and 3′-end integration sites, respectively (arrowheads). Primer sets include excised ϕSa3mw, PCR across the *attP* site, present only in excised prophage DNA (red arrows); intact *hlb*, PCR across the *attB* site, present only in the absence of integrated prophage (purple arrows); integrated ϕSa3mw, PCR across the *attR* site, present at the 3′ end of the integration site (black arrows); ϕSa3mw DNA control, PCR within the ϕSa3mw integrase gene *int* (orange block arrow). (B and C) Time course PCR analysis of prophage excision in MW2 grown with aeration over a 24-h time period in Todd-Hewitt (TH) medium (B) or TH plus 1 mM H_2_O_2_ (C). Genomic DNA control was PCR within the alpha-toxin gene *hla.* (D and E) Time course qPCR analysis of intact *hlb* and integrated ϕSa3mw grown with aeration at designated time points in the presence (E) or absence (D) of 1 mM H_2_O_2_ and expressed as relative fold change from time zero. Results are averages from at least three independent experiments performed in triplicate (means ± SEM). Statistical significance was determined by 2-way ANOVA with Sidak’s multiple-comparison test. (D) Intact *hlb* versus integrated ϕSa3mw trend lines, *P* = 0.018; intact *hlb* and integrated ϕSa3mw time effect, *P* < 0.0001. (E) Intact *hlb* versus integrated ϕSa3mw trend lines, *P* = 0.012; intact *hlb* and integrated ϕSa3mw time effect, not significant; intact *hlb* versus integrated ϕSa3mw at T2h, *P* = 0.049 (*).

To elucidate the temporal dynamics of ϕSa3mw excision and restoration of an intact *hlb* sequence, we PCR screened for the presence of excised ϕSa3mw, intact *hlb*, and integrated ϕSa3mw (Table S1). ϕSa3mw integration occurs by reciprocal site-specific recombination between the bacterial *attB* site and the phage *attP* site. Prophage integration into the *attB* site results in recombinant *att* sites to the left (*attL*) and right (*attR*) of the prophage (36). When a prophage excises, the *attB* and *attP* sites are restored (Fig. 2A). Hence, excised ϕSa3mw was detected by amplification across the *attP* site (present in excised prophage DNA), intact *hlb* was detected by amplification of the *attB* site (present in the absence of integrated prophage), and integrated ϕSa3mw was detected by amplification across the *attR* site (the integrated prophage-bacterial DNA junction). The phage integrase gene *int* was used to assess the presence of ϕSa3mw within the population. The α-toxin gene *hla*, present at a distal site in the chromosome, was used as a genomic DNA control.

In MW2 grown in TH, excised ϕSa3mw was readily detected between 2 and 8 h, corresponding to growth in mid-log to early stationary phase, but not in bacteria from overnight cultures (Fig. 2B). Intact *hlb* was also readily detected from 2 to 5 h of growth, consistent with early and controlled excision events that lead to reintegration in the subpopulation experiencing ϕSa3mw excision. ϕSa3mw is never lost from the population, as integrated prophage and the *int* gene were detected throughout all time points tested (Fig. 2B). Upon exposure to H_2_O_2_, and therefore under bacteriostatic conditions, excised ϕSa3mw and intact *hlb* detection shifted toward earlier time points and over a narrower time window of 1 to 3 h (Fig. 2C). MW2ΔϕSa3mw in TH did not amplify prophage-specific DNA, validating primer specificity (Fig. S3). It is important to note that loss of the prophage does not affect growth under any of the liquid culture conditions tested here (Fig. S3). To confirm that the results accurately inform on the dynamics of ϕSa3mw excision, MW2 with the *int* gene deleted was constructed (MW2Δ*int*) and tested accordingly. Phage integrases are required not only for integration into the chromosome but also for excision (36–38). Hence, ϕSa3mw in the MW2Δ*int* strain will no longer be able to excise from the chromosome. Consistent with the expected function of integrases, excised ϕSa3mw and intact *hlb* were no longer detected by PCR in the MW2Δ*int* strain, while integrated ϕSa3mw was readily detected under all medium conditions tested (Fig. S4).

The PCR screen provided evidence that ϕSa3mw excises during culture conditions, restoring the *hlb* sequence, and remains as a circular phage for at least 8 h. To quantify the frequency at which ϕSa3mw excises from the chromosome, we performed real-time quantitative PCR (qPCR) analysis of intact *hlb* and integrated ϕSa3mw in MW2 grown in the presence or absence of H_2_O_2_. Samples were collected at 2-h intervals for a total of 8 h, and the average prophage excision frequency was calculated as the ratio of intact *hlb* to integrated ϕSa3mw (2^−Δ*CT*^ intact/2^−Δ*CT*^ integrated), as previously described (23). We observed that excision frequencies fluctuated over time during growth in TH with and without H_2_O_2_ (Table 1). In TH, the maximum excision frequency was 8.1 × 10^−4^ (equivalent to 1 in 1,234 cells), and the minimum excision frequency was 2.3 × 10^−4^ (equivalent to 1 in 4,405 cells). In the presence of H_2_O_2_, the maximum and minimum excision frequencies were 18 × 10^−4^ (equivalent to 1 in 560 cells) and 2.4 × 10^−4^ (equivalent to 1 in 4,184 cells), respectively.

**TABLE 1:** Excision frequency of ϕSa3mw from MW2 under liquid culture conditions

Growth medium	Excision frequency at^*a*^:			
	2 h	4 h	6 h	8 h
TH	2.27 ± 0.95 (1:4,405)	8.10 ± 1.81 (1:1,234)	3.07 ± 0.59 (1:3,257)	6.36 ± 2.53 (1:1,572)
TH + 1 mM H_2_O_2_	2.39 ± 0.36 (1:4,184)	11.1 ± 5.78 (1:900)	17.9 ± 3.04 (1:560)	6.80 ± 0.40 (1:1,470)
TSB+G	4.04 ± 3.36 (1:2,475)	10.9 ± 8.01 (1:917)	7.40 ± 5.49 (1:1,351)	1.83 ± 1.19 (1:5,464)
RPMI 1640	1.13 ± 0.15 (1:7,692)	1.89 ± 0.75 (1:5,291)	3.26 ± 0.86 (1:3,067)	0.91 ± 0.07 (1:10,965)

a Excision frequency (×10^−4^) calculated as 2^−Δ*CT*^ intact *hlb*/2^−Δ*CT*^ integrated ϕSa3mw. Values are means ± SEM. Cell ratios are 1/excision frequency. Excision frequency at T0h (i.e., overnight growth in TH), 1.38 × 10^−4^ ± 0.82 × 10^−4^ (1:7,246).

To establish if ϕSa3mw excision was induced or repressed by culture conditions over time, the qPCR results of intact *hlb* and integrated ϕSa3mw were expressed as relative fold change from time zero (2^−ΔΔ*CT*^). Both conditions led to significant increases in intact *hlb* compared to levels for integrated ϕSa3mw (Fig. 2D and E). However, in TH, the relative fold change of intact *hlb* peaked around 4 h and remained elevated until the 6-h time point (Fig. 2D). There was an increase in both intact *hlb* and integrated ϕSa3mw from the 2-h to 4-h time points that we interpreted as incomplete normalization by the gyrase gene (*gyrA*). These results were consistent throughout multiple experiments, yet it likely represents bacterial growth. In contrast, in the presence of H_2_O_2_, the relative fold change of intact *hlb* significantly peaked at 2 h and decreased over time (Fig. 2E). The relative fold change of integrated ϕSa3mw did not significantly change over time under either condition (Fig. 2D and E). These results are consistent with broader but less frequent excision events in TH versus narrower but more frequent excision events in the presence of H_2_O_2_.

### Tissue culture medium RPMI 1640 does not promote ϕSa3mw excision.

**FIG 3:**
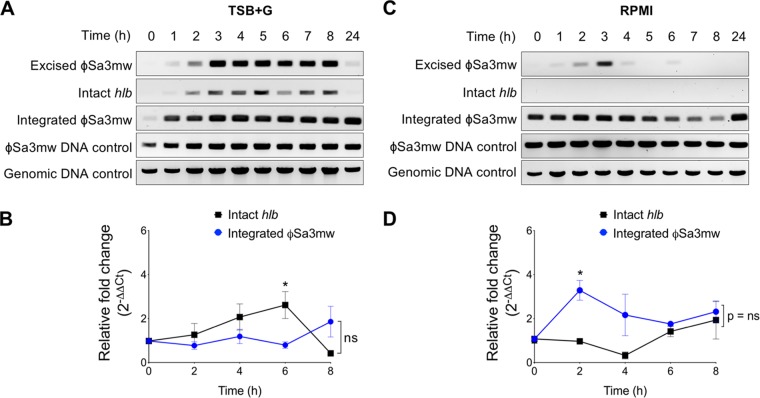
Detection of excised ϕSa3mw is greatly reduced in bacteria grown in RPMI tissue culture medium. (A and C) PCR analysis of prophage excision during MW2 planktonic growth over a 24-h time period in tryptic soy broth (TSB) supplemented with 2% glucose and 2% NaCl (TSB+G) (A) or tissue culture medium RPMI 1640 buffered with 10 mM HEPES (C). Excised ϕSa3mw, PCR across the *attP* site, present only in excised prophage DNA; intact *hlb*, PCR across the *attB* site, present only in the absence of integrated prophage; integrated ϕSa3mw, PCR across the *attR* site, present at the 3′ end of the integration site; ϕSa3mw DNA control, PCR within the ϕSa3mw integrase gene *int*; genomic DNA control, PCR within the alpha-toxin gene *hla*. (B and D) qPCR analysis of intact *hlb* and integrated ϕSa3mw at specific time points in TSB+G (B) or RPMI 1640 (D) and expressed as relative fold change from time zero. Results are averages from at least three independent experiments performed in triplicate (means ± SEM). Statistical significance was determined by 2-way ANOVA with Sidak’s multiple-comparison test. (B) Intact *hlb* versus integrated ϕSa3mw trend lines, not significant (ns); intact *hlb* and integrated ϕSa3mw time effect, not significant (ns); intact *hlb* versus integrated ϕSa3mw at the 6-h time point (T6h), *P* = 0.01 (*). (D) Intact *hlb* versus integrated ϕSa3mw trend lines, not significant (ns); intact *hlb* and integrated ϕSa3mw time effect, *P* = 0.005; intact *hlb* versus integrated ϕSa3mw at T2h, *P* = 0.01 (*).

To further characterize the conditional excision of ϕSa3mw during planktonic growth, we performed PCR and qPCR analyses of MW2 growing aerobically in biofilm-promoting medium (tryptic soy broth supplemented with 2% glucose and 2% NaCl; TSB+G) and tissue culture medium (buffered RPMI 1640), as these conditions are commonly used to assess virulence of S. aureus in various *in vitro* assays. The excision profile of MW2 grown in TSB+G showed distinct ϕSa3mw excision events during log growth that correlated with detection of intact *hlb* by PCR (Fig. 3A). Again, excised ϕSa3mw and intact *hlb* were only faintly detected at 24 h (Fig. 3A). qPCR analysis of intact *hlb* and integrated ϕSa3mw showed a maximum excision frequency of 11 × 10^−4^ (equivalent to 1 in 917 cells) and minimum excision frequency of 1.8 × 10^−4^ (equivalent to 1 in 5,464 cells) (Table 1). Relative fold change from time zero (2^−ΔΔ*CT*^) showed a significant increase of intact *hlb* at 6 h, with no significant changes of integrated ϕSa3mw over time (Fig. 3B). Consistent with these results, growth of MW2 containing β-toxin–sGFP in TSB+G leads to significantly higher levels of β-toxin–sGFP after 6 h of growth (Fig. S2).

Surprisingly, MW2 growth in RPMI 1640 vastly diminished detection of excised ϕSa3mw and eliminated detection of intact *hlb*, as assessed by PCR, while integrated ϕSa3mw was readily detected at all time points (Fig. 3C). These results are consistent with overall lower excision frequencies during growth in RPMI 1640 (maximum excision of 3.3 × 10^−4^, equivalent to 1 in 3,067 cells, and minimum excision of 0.91 × 10^−4^, equivalent to 1 in 10,965 cells) (Table 1). Relative fold change from time zero (2^−ΔΔ*CT*^) confirmed these results, with no significant increases of intact *hlb* observed over time (Fig. 3D). Instead, the trend was reversed where the relative fold change of integrated ϕSa3mw significantly increased in early exponential phase (2 h) before normalizing at later time points. Similarly, detection of β-toxin–sGFP from MW2 grown in RPMI 1640 dropped below basal levels that had resulted from overnight growth in TH (Fig. S2). Altogether, these results indicate that growth in TSB+G promotes ϕSa3mw excision while growth in RPMI 1640 suppresses it, providing further evidence of the dynamic nature of ϕSa3mw under different culture conditions.

### Biofilms promote ϕSa3mw excision.

**FIG 4:**
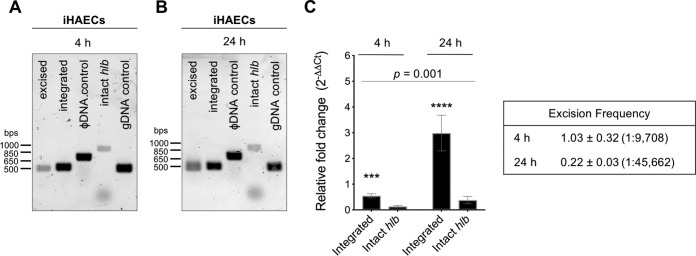
ϕSa3mw excision is not promoted during infection of immortalized human aortic endothelial cells (iHAECs). iHAECs were infected at an MOI of 100 for 1 h, washed, and cultured for an additional 4 h and 24 h. (A and B) PCR analysis of prophage excision during iHAEC infection at 4 h (A) or 24 h (B). Excised, PCR across the ϕSa3mw *attP* site, present only in excised prophage DNA; integrated, PCR across the *attR* site, present at the 3′ end of the integration site; ϕSa3mw DNA control, PCR within the ϕSa3mw integrase gene *int*; intact *hlb*, PCR across the *attB* site, present only in the absence of integrated prophage; genomic DNA control (gDNA), PCR within the alpha-toxin gene *hla*. (C, left) qPCR analysis of integrated ϕSa3mw and intact *hlb* from iHAEC culture lysates at 4 h and 24 h and expressed as relative fold change from overnight cultures of MW2 grown in TH. (Right) Excision frequencies (×10^−4^) at 4 h and 24 h calculated as 2^−Δ*CT*^ intact *hlb*/2^−Δ*CT*^ integrated ϕSa3mw. Cell ratios were calculated as 1/excision frequency. Excision frequency at T0h, 1.38 × 10^−4^ ± 0.82 × 10^−4^ (1:7,246). Results are averages from at least three independent experiments performed in triplicate (means ± SEM). Statistical significance was determined by 2-way ANOVA with Sidak’s multiple-comparison test. (C) Integrated ϕSa3mw versus intact *hlb*, *P* = 0.0002 (4 h) (***) and *P* < 0.0001 (24 h) (****). Integrated ϕSa3mw and intact *hlb* time effect, *P* = 0.001.

β-Toxin-producing S. aureus subsets are more prevalent in heart vegetations in a rabbit model of infective endocarditis (20). In endocarditis, S. aureus colonizes the heart endothelium and forms a tissue biofilm (vegetation) composed of plasma factors and bacteria. We addressed whether S. aureus interaction with endothelial cells or growth in a static biofilm could induce ϕSa3mw excision. For this, immortalized human aortic endothelial cells (iHAECs) grown to confluence were infected with MW2 at a multiplicity of infection (MOI) of 100. After 1 h, cells were washed to remove nonadherent bacteria and adherent bacteria were allowed to propagate. Culture lysates (cells and media) were collected at 4 h and 24 h. Excised ϕSa3mw and intact *hlb* could be detected by PCR at both time points (Fig. 4A and B). However, the relative fold change (2^−ΔΔ*CT*^) of integrated ϕSa3mw was significantly higher than that of intact *hlb*, resulting in excision frequencies of 1.0 × 10^−4^ (equivalent to 1 in 9,709 cells) and 0.22 × 10^−4^ (equivalent to 1 in 45,662 cells) at 4 h and 24 h, respectively (Fig. 4C). These results indicate that ϕSa3mw is preferentially maintained as an integrated prophage in MW2 grown on endothelial cells in culture.

**FIG 5:**
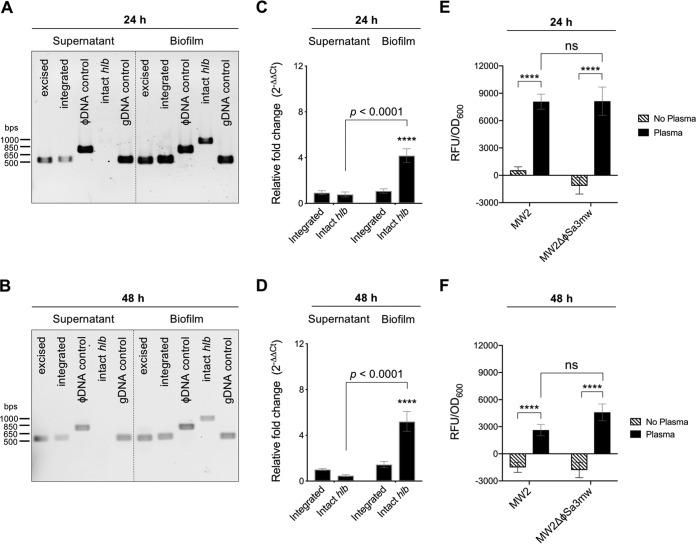
ϕSa3mw excises during biofilm growth. (A and B) PCR analysis of prophage excision during growth in a stationary biofilm in tryptic soy broth supplemented with 2% glucose and 2% NaCl (TSB+G) for 24 h (A) or 48 h (B) and respective supernatants. Excised, PCR across the ϕSa3mw *attP* site, present only in excised prophage DNA; integrated, PCR across the *attR* site, present at the 3′ end of the integration site; ϕSa3mw DNA control, PCR within the ϕSa3mw integrase gene *int*; intact *hlb*, PCR across the *attB* site, present only in the absence of integrated prophage; genomic DNA control (gDNA), PCR within the alpha-toxin gene *hla*. (C and D) qPCR analysis of integrated ϕSa3mw and intact *hlb* from bacterial biofilms and supernatants at 24 h (C) and 48 h (D) and expressed as relative fold change from overnight cultures of MW2 grown in TSB+G. (E and F) Detection of β-toxin–sGFP from MW2 or MW2ΔϕSa3mw that was grown in biofilms (black bars) or planktonic (patterned bars) after 24 h (E) or 48 h (F). Results are averages from at least three independent experiments performed in triplicate (means ± SEM). Statistical significance was determined by 2-way ANOVA with Sidak’s multiple-comparison test. (C and D) Integrated ϕSa3mw in supernatants versus in biofilms at both 24 h and 48 h, not significant; intact *hlb* in supernatants versus in biofilms at both 24 h and 48 h, *P* < 0.0001; integrated ϕSa3mw versus intact *hlb* in biofilms at both 24 h and 48 h, *P* < 0.0001 (****). (E and F) MW2 RFU/OD_600_ in biofilms versus planktonic at both 24 h and 48 h, *P* < 0.0001 (****); MW2ΔϕSa3mw RFU/OD_600_ in biofilms versus planktonic at both 24 h and 48 h, *P* < 0.0001 (****); MW2 versus MW2ΔϕSa3mw RFU/OD_600_ in biofilms, not significant; MW2 versus MW2ΔϕSa3mw RFU/OD_600_ planktonic, not significant.

We then addressed whether growth in a biofilm induces ϕSa3mw excision. MW2 was grown in human plasma-coated wells in TSB+G for 24 h or 48 h at 37°C with 5% CO_2_. Since TSB+G induces ϕSa3mw excision during growth in liquid culture, biofilm supernatants containing planktonic bacteria and bacterial biofilms were collected separately. At both time points, excised ϕSa3mw was readily detected by PCR in both planktonic and biofilm bacteria. However, intact *hlb* was only detected in bacteria recovered from biofilms (Fig. 5A and B). qPCR analysis of bacteria recovered from supernatants or biofilms were consistent with these results, where the relative fold change (2^−ΔΔ*CT*^) of intact *hlb* was significantly increased in bacteria growing in biofilms (Fig. 5C and D). The excision frequencies between planktonic bacteria at 24 h (1.6 × 10^−4^; equivalent to 1 in 6,329) and at 48 h (0.85 × 10^−4^; equivalent to 1 in 11,820) and those growing in a biofilm at 24 h (10 × 10^−4^; equivalent to 1 in 980) and at 48 h (7.6 × 10^−4^; equivalent to 1 in 1,314) confirmed the differential excision of ϕSa3mw in stationary biofilms (Table 2).

**TABLE 2:** Excision frequency of ϕSa3mw from MW2 biofilms

Cell population	Excision frequency at^*a*^:	
	24 h	48 h
Supernatant	1.58 ± 0.41 (1:6,329)	0.85 ± 0.26 (1:11,820)
Biofilm	10.2 ± 3.91 (1:980)	7.61 ± 3.45 (1:1,314)

a Excision frequency (×10^−4^) calculated as 2^−Δ*CT*^ intact *hlb*/2^−Δ*CT*^ integrated ϕSa3mw. Values are means ± SEM. Cell ratios are 1/excision frequency. Excision frequency at T0h (i.e., overnight growth in TSB+G), 3.67 × 10^−4^ ± 0.33 × 10^−4^ (1:7,246).

To assess whether ϕSa3mw excision during growth in a biofilm results in increased β-toxin production, biofilm assays were performed with MW2 β-toxin–sGFP and the phage-cured variant also encoding the translational fusion (MW2ΔϕSa3mw β-toxin–sGFP). β-toxin–sGFP fluorescence was first normalized to the optical density of the resuspended biofilm, and then fluorescence from the negative control was subtracted (biofilm formed by S. aureus not expressing GFP). Indeed, growth in a biofilm resulted in equivalent levels of β-toxin–sGFP detected from MW2 and MW2ΔϕSa3mw at 24 h and 48 h (Fig. 5E and F). We also assessed β-toxin production in the biofilm assay but in the absence of plasma (non-biofilm-inducing condition similar to planktonic growth in biofilm supernatant). After normalization to optical density, fluorescence from the negative control was subtracted (S. aureus not expressing GFP grown without plasma). Interestingly, β-toxin–sGFP was detected at or below the threshold in cultures grown in the absence of plasma, suggesting preferential β-toxin production within biofilms (Fig. 5E and F). Finally, we assessed if increased ϕSa3mw excision in biofilms led to the release of phage particles. Again, no phage particles were detected from planktonic or biofilm cultures of MW2 as assessed by TEM (data not shown). These results provide evidence that growth in a biofilm induces ϕSa3mw excision with concomitant β-toxin production. Hence, ϕSa3mw excision could contribute to the appearance of β-toxin-producing subsets during infection.

## DISCUSSION

Staphylococcus aureus is a highly successful opportunistic bacterial pathogen that colonizes the mucosal surfaces of approximately 30% of humans (3, 39). β-Toxin is a sphingomyelinase hemolysin, encoded by most S. aureus strains, that promotes S. aureus mucosal colonization in humans and, in rabbit models, biofilm formation on heart valves (vegetations) and lung granulomas (11, 40–42). However, the contribution of β-toxin to S. aureus pathogenesis remained unclear due to integration of the ϕSa3int family of prophages into the β-toxin structural gene *hlb* in the vast majority of human isolates (9, 10). Hence, most strains are reported to not produce β-toxin. Recently, multiple studies provided evidence that phage loss, resulting in β-toxin production, increases specifically during infections (11, 19, 20, 22, 43, 44). In fact, ϕSa3int can be induced during active and chronic infections as a result of host pressure or antibiotic therapy. Phage regulatory switches (phage-RSs) are a newly described form of active lysogeny, where prophages cooperate with their host for expression of disrupted genes or restoration of regulatory sequences (35). This contrasts with latent lysogeny, where the prophage senses danger (usually conditions that damage DNA), excises from the chromosome, and launches into its lytic cycle, producing many virions before lysing the bacterial cell (45). A central question is do the ϕSa3int prophages function as phage-RSs for the conditional expression of β-toxin? Here, we evaluated this dynamic under *in vitro* conditions relevant to S. aureus pathogenesis.

We provide evidence that β-toxin production occurs by a mechanism of active lysogeny and not classic latent lysogeny. H_2_O_2_-induced oxidative stress, a common phage-inducing signal (33, 34, 46), results in early phage excision events and in a significant but transient increase in β-toxin production that is not due to changes in *hlb* promoter activity, as the promoter remains active once induced. Furthermore, the concomitant detection of extrachromosomal, circular ϕSa3mw and an intact *hlb* sequence under bacteriostatic conditions is consistent with induction of ϕSa3mw excision and not enrichment or selection of phage-cured variants, as could happen during growth or during infection. However, we have no evidence that ϕSa3mw excision results in host cell lysis or the release of infectious virions. No plaques are observed after transduction with MW2 supernatants from H_2_O_2_- or mitomycin C-treated cultures, and no phage particles are observed by TEM in these supernatants. In stark contrast, culture supernatants from the S. aureus lytic phage ϕ11 propagated in RN4220 contain many virions readily visualized by TEM. Instead, the data support the hypothesis that ϕSa3mw functions as a reversible switch, where the prophage reinserts into *hlb* to reestablish lysogeny, terminating β-toxin production. For instance, detection of an intact *hlb* sequence is finite, and the time-dependent interruption of the sequence is consistent with a significant reduction in β-toxin–sGFP detected in culture. Our results are also consistent with previous reports that ϕSa3mw is capable of replication (6, 8), since excised ϕSa3mw is readily detected past the time points of maximal detection of intact *hlb*.

In active lysogeny, the prophage responds to an environmental cue and excises. Hence, we addressed whether ϕSa3mw excision and β-toxin production were differentially induced under culture conditions used to study S. aureus pathogenesis *in vitro*. S. aureus aerobic growth in rich culture medium (TH), in biofilm-promoting medium (TSB+G), and in tissue culture medium (RPMI 1640) exhibits almost identical growth kinetics. However, β-toxin–sGFP is differentially detected under those conditions, with a trend that is consistent with the relative fold change of intact *hlb* over time. S. aureus growth in TH and TSB+G results in production of lower levels of β-toxin–sGFP that is both induced and terminated over time but extending to at least 8 h of growth. Interestingly, TH and TSB+G are not significantly different in inducing maximum detection of β-toxin–sGFP but rather differ in the time points where the maximum fluorescence is induced. In TH, maximum detection of β-toxin–sGFP is at 4 to 6 h, while in TSB+G it is at 6 to 8 h. This broader, albeit lower, expression of β-toxin in TH and TSB+G correlates with detection of an intact *hlb* sequence over similar time frames. Surprisingly, aerobic growth in RPMI 1640 suppresses ϕSa3mw excision, as no intact *hlb* was detected during growth. There is some detection of excised ϕSa3mw at early time points that is likely carryover of excision events that occurred during overnight culture in TH, resulting in phage replication, but then rapidly lost over time during growth in RPMI. These results emphasize the ability of ϕSa3mw to respond to environmental cues, from excising during bacteriostatic conditions to remaining integrated or differentially excising during growth in exponential phase.

Recently, we showed that MW2 β-toxin^+^ variants are enriched in vegetations collected from rabbit heart valves in experimental infective endocarditis (20). Vegetations are, in essence, biofilms formed on the endothelium (40, 47). Thus, we further examined ϕSa3mw excision in cultured endothelial cells and during growth in a biofilm, as these *in vitro* conditions are relevant to infective endocarditis. S. aureus intimately associates with endothelial cells, as the endothelium acts as a barrier to invading bacteria, as an adhesive and cytotoxic target for bacteria, and as an immune regulatory cell (48–51). However, S. aureus interactions with endothelial cells in culture preferentially maintain ϕSa3mw as an integrated prophage, since excision occurs at very low frequencies. In stark contrast, MW2 growth in a biofilm induces ϕSa3mw excision, consistent with a significant increase in intact *hlb* compared to planktonic bacteria in biofilm supernatants. Furthermore, bacteria growing in a biofilm induce β-toxin–sGFP production to levels similar to that of the isogenic non-lysogen strain. Yet again, virions are not released into the biofilms. Interestingly, transcriptome analysis of S. aureus biofilm cells found that the biofilm environment upregulates responses to oxidative stress (52). It is therefore possible that in our studies, the increased ϕSa3mw excision events in response to both H_2_O_2_ and biofilm growth result from similar mechanisms. In terms of endocarditis, the selective and differential induction of ϕSa3mw excision suggests that β-toxin largely promotes vegetative growth and persistence.

Altogether, we provide evidence that ϕSa3mw (the ϕSa3int prophage in MW2) functions as a phage-RS. ϕSa3mw preferentially excises from *hlb* in response to oxidative stress and during biofilm growth but is not induced by human endothelial cells in culture. ϕSa3mw excision leads to a time-dependent restoration of an intact *hlb* and β-toxin production. However, it is not strictly dependent on bacterial growth, as it can be detected under bacteriostatic conditions. Finally, ϕSa3mw excision does not result in the release of infectious phage particles, indicating that host cell lysis is not likely to occur. The basic physiology of the ϕSa3int family of prophages remains poorly understood. Phage dynamics and the fate of the excised phage (whether it reintegrates, is lost, is retained as a nonreplicating episome, or becomes lytic) are likely dictated by the conditions encountered in the mammalian host. Future studies will focus on understanding this three-partner interaction, as it is not only of fundamental interest but also critical when designing intervention strategies and therapeutics.

S. aureus infections are the result of a myriad of virulence factors acting in a coordinated fashion. How and when these virulence factors contribute to infection are the subject of ongoing research. β-Toxin generally has been ignored as a major virulence factor due to negative conversion by integration of the ϕSa3int prophage. ϕSa3int prophages encode several superantigens (SEA, SE*l*-K, and SE*l*-Q) and innate immune modulators (SAK, SCIN, and CHIP) that were presumed to be more advantageous to S. aureus virulence than β-toxin (9). Our findings with ϕSa3mw in S. aureus MW2 provide evidence that β-toxin is produced by the controlled excision of the ϕSa3int prophage. ϕSa3int excision during active disease highlights a dynamic and perhaps more sophisticated S. aureus-prophage interaction, where ϕSa3int provides a novel regulatory mechanism for conditional virulence gene expression and, thus, increased fitness.

## MATERIALS AND METHODS

### Media and reagents.

Bacto Todd-Hewitt broth (TH) and Bacto tryptic soy broth (TSB) were purchased from Becton, Dickinson (Sparks, MD). Gibco RPMI 1640 and 0.25% trypsin-EDTA were purchased from Life Technologies Corporation (Grand Island, NY). Pooled human plasma was purchased from Innovative Research, Inc. (Novi, MI). Power SYBR green master mix was purchased from Applied Biosystems and used according to the manufacturer’s instructions (Foster City, CA). Mitomycin C (no. M4287), lysostaphin (no. L7386), and mutanolysin (no. M9901) were purchased from Sigma-Aldrich (St. Louis, MO). Goat anti-rabbit antibody–IRDye 680LT and goat anti-rabbit antibody–IRDye 800CW were purchased from LI-COR Biosciences (Lincoln, NE). Anti-GFP antibody was a kind gift of David Weiss, University of Iowa. Anti-β-toxin antibody was produced in-house. All other enzymes, polymerases, and Gibson Assembly master mix were purchased from New England Biolabs (Beverly, MA) and used according to the manufacturer’s instructions unless otherwise noted. All oligonucleotides were purchased from Integrated DNA Technologies (IDT; Coralville, IA).

### Bacterial strains, plasmids, and growth conditions.

Staphylococcal strains were utilized from low-passage-number stocks. Unless otherwise indicated, staphylococcal strains were maintained at 37°C with aeration (220 rpm) in TH broth. Strains and plasmids used in this study are listed in Table 3. All plasmids were maintained in E. coli DH5α. Plasmids were moved into S. aureus RN4220 by electroporation using a 2-mm pulse cuvette and then shuttled into the clinical isolate MW2 or prophage-cured MW2ΔϕSa3mw by generalized transduction with bacteriophage ϕ11 (53). Carbenicillin (100 μg ml^−1^) was used to maintain all plasmids in E. coli. Chloramphenicol (20 μg ml^−1^) or erythromycin (20 μg/ml^−1^) was used to maintain pJB38 or pCE104, respectively, in S. aureus.

**TABLE 3:** Strains and plasmids

Strain or plasmid	Genotypic description	Reference or source
Strains		
RN4220	Restriction-deficient cloning strain	20
MW2	Wild-type S. aureus strain from a pneumonia clinical isolate containing ϕSa3mw	20
MW2ΔϕSa3mw	MW2 naturally cured of ϕSa3mw	20
MW2Δ*int*	MW2 containing an in-frame deletion of the ϕSa3mw integrase gene *int*	This study
Plasmids		
pCE104	Shuttle vector containing pE194 and pUC18	60
pPMT2	pCE104 containing the MW2 P*_hlb_*	This study
pPMT4	pPMT2 containing sGFP under the MW2 P*_hlb_* control	This study
pTH100	pJB38-NWMN29-30 containing sGFP	54
pJB38	Temp-sensitive shuttle vector for S. aureus chromosomal integration	61
pKK65	pJB38 modified for Golden Gate assembly	This study

### Construction of *hlb* promoter reporter (P*_hlb_-sgfp*).

PCR was performed with Phusion polymerase to amplify the *hlb* promoter from MW2 cell lysate using primer set hlb_prom_Forward/hlb_prom_Reverse. The PCR product was digested with XmaI and KpnI and ligated into pCE104 digested by the same enzymes, creating pPMT2. A second PCR was performed to amplify superfolding GFP (sGFP) from pTH100 (Addgene plasmid number 84458) using primer set sGFP_For/gfpnew_R. This PCR product was digested with EcoRI and KpnI and ligated into pPMT2 digested by the same enzymes. The plasmid, pPMT4, contains the *hlb* promoter driving expression of sGFP.

### Construction of *hlb-sgfp* C-terminal translational fusion (β-toxin–sGFP) reporter.

A gene block (gBlock) was purchased from IDT containing the 3′ catalytic region of *hlb* (MW_RS10565) fused to sGFP with the stop codon moved immediately after the sGFP gene. The gBlock was digested with EcoRI and KpnI and ligated into pJB38, digested by the same enzymes, to produce a pJB38 intermediate. Concurrently, PCR was used to amplify downstream of *hlb* with primer set gBlock_DownstreamF/gBlock_DownstreamR. The pJB38 intermediate was digested with KpnI and the PCR product inserted by Gibson Assembly. The final construct was moved into MW2 as described above. The construct was then integrated into the chromosome as previously described (54).

### Construction of GFP dropout vector pKK65.

To create a Golden Gate cloning vector, the native pJB38 BsaI cut site was eliminated by mutagenesis with primer set BsaIdelF/BsaIdelR using an NEB Q5 site-directed mutagenesis kit (E0554S). A gBlock was purchased from IDT containing the sarA-P1 promoter fused to sGFP with a lambda t0 terminator and flanking BsaI restriction sites. The gBlock was amplified using Phusion polymerase with primer set pJB38_sarAP1-sGFPF/pJB38_sarAP1-sGFPR and subsequently inserted into pJB38, previously digested with SacI, using Gibson Assembly. The resulting plasmid was named pKK65.

### Construction of MW2Δ*int* strain.

PCR was performed with Phusion polymerase to amplify the upstream and downstream regions immediately flanking *int* (MW_RS10560) from MW2 cell lysate with the primer sets intKOUpF/intKOUpR and intKODnF/intKODnR. Golden Gate assembly was used to ligate the PCR product into pKK65 using T4 DNA ligase and BsaI-HFv2 (55). The resulting plasmid was shuttled into MW2 and integrated into the chromosome as previously described (54). Colonies were selected for on TH agar with 0.1 μg ml^−1^ anhydrotetracycline. The in-frame deletion was detected by PCR, and positive selections were verified by sequencing.

### Fluorescence reporter activity.

Overnight TH cultures of bacteria containing the P*_hlb_-sgfp* or β-toxin–sGFP reporter were subcultured at 1 × 10^7^ cells ml^−1^ into TH with and without 1 mM H_2_O_2_, TSB+G, or RPMI 1640 in black 96-well plates (Nunc, ThermoFisher Scientific, Waltham, MA) (56). Experiments with an empty vector control (MW2 containing pCE104) were also conducted to account for autofluorescence. Fluorescence was measured every hour in a TECAN Infinite M200 Pro (Tecan Group Ltd., Männedorf, Switzerland) with the following settings: excitation at 436 nm, emission at 564 nm, Z position at 21,374 μm, and optimal gain. A minimum of three biological replicates were conducted for each condition. Readings from three wells per biological replicate were averaged, empty vector control readings were subtracted, and data were normalized (RFU_sample_ − RFU_min_)/(RFU_max_ − RFU_min_) for every run, where RFU is the relative fluorescence units, min is the minimum RFU value for a given well, and max is the maximum RFU for a given well.

### SDS-PAGE and Western blotting.

Cultures were grown overnight and centrifuged (1,000 × *g*, 5 min), and then the supernatant was passaged through a 0.45-μm filter. Samples were mixed with sample buffer, boiled for 10 min, and electrophoresed on a 4% to 20% gradient Mini-PROTEAN TGX stain-free gel (Bio-Rad Laboratories, Hercules, CA) at 160 V for 40 min along with a Precision Plus protein dual-color standard (Bio-Rad). This was then transferred to a GE polyvinylidene fluoride (PVDF) membrane (Suez Water Technologies, Trevose, PA) by electroblotting at 200 V for 2 h at 4°C. The membrane was blocked with 5% nonfat dry milk in phosphate-buffered saline (PBS; 2 mM NaH_2_PO_4_, 5.7 mM Na_2_HPO_4_, 0.1 M NaCl, pH 7.4) for 1 h at room temperature and then incubated with rabbit polyclonal anti-toxic shock syndrome toxin 1 antibody (1:1,000) overnight at 4°C as an attempt to quench interfering signal from S. aureus protein A. The membrane was washed (PBS with 0.1% Tween 20) and then incubated with goat anti-rabbit antibody–IRDye 800CW (1:10,000) for 1 h at room temperature. After washing, the membrane was incubated with either rabbit polyclonal anti-β-toxin antibody (1:1,000) or anti-GFP antibody (1:1,000) overnight at 4°C. The membrane was washed and then incubated with goat anti-rabbit antibody–IRDye 680LT (1:10,000) for 1 h at room temperature. Blots were visualized using an Odyssey CLx (LI-COR).

### Mitomycin C treatment.

Overnight cultures were subcultured 1:200 into fresh TH and incubated to mid-log growth, after which 1 μg ml^−1^ mitomycin C was used to induce ϕSa3mw excision for 4 h. Samples were then further processed depending on the experiment as described below.

### Plaque assay.

Plaque assays were performed as previously described (6, 40), using MW2 grown overnight in TH with and without 1 mM H_2_O_2_ or MW2 treated with mitomycin C as described above. Indicator strains were MW2ΔϕSa3mw and RN4220. Prophages were isolated from MW2 cultures by centrifugation (1,000 × *g*, 5 min), followed by passage through a 0.45-μm filter. Indicator strains were grown overnight in TH, and 10 μl or 100 μl of growth was plated on TH agar and allowed to air dry at room temperature. Ten or 20 μl of filtered supernatant was spotted onto the indicator strains and allowed to absorb for 15 min at room temperature. Plates were incubated at 37°C and checked for plaques after 24 h.

### Transmission electron microscopy.

To prepare prophage samples for TEM, bacteria collected from the various tested conditions were pelleted by centrifugation (1,000 × *g*, 5 min, 4°C) and passed through a 0.45-μm filter. Prophage were then isolated as previously described (6). Briefly, samples were treated with lysostaphin (40 μg ml^−1^), mutanolysin (100 U ml^−1^), DNase I (2 U), RNase A (4 μg ml^−1^), and one cOmplete, mini protease inhibitor tablet (Roche, Mannheim, Germany) for 2 h at 37°C. Samples were centrifuged (50,000 × *g*, 1 h, 4°C) and resuspended in 500 μl TM buffer (10 mM Tris-HCl, 10 mM MgCl_2_, pH 8.5). Samples were placed on a carbon-coated grid and stained with 1% uranyl acetate for 30 s (ϕ11) or 2 min (ϕSa3mw). All samples were analyzed using a JEOL JEM-1230 transmission electron microscope at the University of Iowa Central Microscopy Research Facility.

### ϕSa3mw and MW2 PCR screen.

Overnight cultures of MW2 or MW2ΔϕSa3mw were subcultured into 50 ml of TH with and without 1 mM H_2_O_2_, TSB+G (TSB supplemented with 2% glucose and 2% NaCl), or Gibco RPMI 1640 buffered with 10 mM HEPES in a 250-ml flask so that the starting concentration was 1 × 10^7^ cells ml^−1^. Samples were taken hourly for 8 h, followed by a final sample at 24 h. At the time of sample collection, the samples were pelleted by centrifugation (16,000 × *g*, 1 min), the supernatant was removed, and the pellet was stored at −20°C. Pellets were resuspended in lysis buffer (20 mM Tris-HCl [pH 8.5], 1% Triton X-100, 2 mM EDTA) and incubated for 15 min at 94°C. DNA concentration of the lysate was determined by NanoDrop (ThermoFisher Scientific, Waltham, MA) and then adjusted to match the lowest concentration. PCR was performed with Phusion polymerase to amplify excised ϕSa3mw (*attP* site) with primer set Sa3ExcisF/Sa3ExcisR, integrated ϕSa3mw (*attR* site) with primer set Sa3InF/Sa3InR, intact *hlb* (*attB* site) with primer set hlbscreenF/hlbscreenR, ϕSa3mw integrase (*int*) with primer set intF/intR, and *hla* (MW_RS05625) with primer set hlaF/hlaR. Primer set seaF/seaR was used to detect *sea* (MW_RS10305) in the MW2Δ*int* strain. PCR products were visualized on 1% Tris-acetate-EDTA (TAE)–agarose gels stained with ethidium bromide (Bio-Rad). Electrophoresis was carried out at 130 V for 40 min in 1× TAE and visualized with UV transillumination using a Chemidoc Touch imaging system (Bio-Rad).

### qPCR.

MW2 was grown under various conditions as described above. To extract genomic DNA, bacteria were pelleted and lysed in buffer (3 mg ml^−1^ lysozyme, 0.5 mg ml^−1^ lysostaphin, 1% Triton X-100 in Tris-EDTA buffer) for 1 h at 37°C. Lysates were incubated for 30 min at 70°C and then further processed using a Qiagen DNeasy blood and tissue kit by following the manufacturer’s instructions (Qiagen, Hilden, Germany). Samples were eluted with nuclease-free water, and the DNA concentration was quantified by NanoDrop. qPCR was performed using the Power SYBR green master mix with specific primers designed using OligoArchitect Online (see Table S1 in the supplemental material). A minimum of three biological replicates were conducted for each experimental condition. All qPCRs were performed with three replicates in a total volume of 25 μl, consisting of 30 ng of target DNA and 200 nM each primer. Amplification and detection were carried out on a Stratagene Mx3005P (Agilent, Santa Clara, CA). Data were analyzed by the 2^−ΔΔ*CT*^ method described by Livak and Schmittgen (57). Δ*C_T_* was calculated for each target (equation 1), where *C_T_* is the threshold cycle for target amplification, TOI is the target of interest, and *gyrA* is the housekeeping gene used for normalization. Δ*C_T_* was averaged for each target and ΔΔ*C_T_* calculated (equation 2). Fold change was calculated as 2^−ΔΔ*CT*^.

(1) $$\Delta C_{T}=\;C_{T\;(\mathrm{TOI})}-C_{T\;(\mathrm{gyrA})}$$

(2) $$\Delta \Delta C_{T}=\Delta C_{T}{}_{{}_{\mathrm{Time}x}}-\Delta C_{T}{}_{{}_{\mathrm{Time}0}}$$

### Endothelial cell culture assays.

Immortalized human aortic endothelial cells (iHAECs) were generated and maintained as previously described (58). All experiments were conducted using iHAECs at passages between 4 and 10 from a single clone. Twenty-four-well tissue culture plates coated with 1% gelatin were seeded with 50,000 cells well^−1^ and grown to confluence. Wells were washed, and cells were infected with overnight cultures of MW2 or MW2ΔϕSa3mw at an MOI of 100. Infections occurred for 1 h at 37°C, 5% CO_2_, after which media were collected and bacteria stored as described above. iHAECs were washed and fresh medium was added, after which bacteria were allowed to propagate for another 4 and 24 h. After propagation, all contents of the wells were collected by lysing iHAECs with 0.25% trypsin-EDTA and 0.25% Triton X-100. Lysates were stored as described above.

### Biofilm growth.

Assays were conducted as previously described (59). Briefly, MW2 or MW2ΔϕSa3mw was grown overnight with aeration at 37°C in TSB+G (TSB supplemented with 2% glucose and 2% NaCl). Overnight cultures were subcultured in TSB+G and used to inoculate 96-well plates previously coated with 20% pooled human plasma at a final concentration of 1 × 10^7^ cells well^−1^. Plates were incubated for the designated amount of time at 37°C in 5% CO_2_. The media were collected and cells pelleted and stored as described above. Wells were then washed with PBS and the biofilms were resuspended with PBS, pelleted, and processed as described above for screening PCR and qPCR. To detect β-toxin–sGFP from biofilms, experiments were conducted as described above using MW2 and MW2ΔϕSa3mw containing the *hlb-sgfp* translational fusion. After incubation, the well contents were resuspended for homogeneity and fluorescence was measured with a TECAN Infinite M200 Pro as described above. Absorbance (optical density at 600 nm [OD_600_]) was measured after fluorescence. A minimum of three biological replicates with three technical replicates were conducted for each condition. Fluorescence per biological replicate was normalized to OD_600_, followed by subtraction of fluorescence from the empty vector control, and data are presented as RFU/OD_600_.

### Statistical analyses.

All statistical analyses were performed by a two-way analysis of variance (ANOVA) using the Šídák method to correct for multiple comparisons with a 0.05 significance cutoff using GraphPad Prism software (San Diego, CA).

## Supplementary Material

Supplemental file 1
