# Supplementary material for: ϕSa3mw Prophage as a Molecular Regulatory Switch of Staphylococcus aureus β-Toxin Production
Source: J Bacteriol. 2019 Jun 21;201(14):e00766-18. doi: 10.1128/JB.00766-18 (PMC6597384; doi:10.1128/JB.00766-18)
Supplement: Supplemental file 1 [file JB.00766-18-s0001.pdf]

Supplemental Figure 1

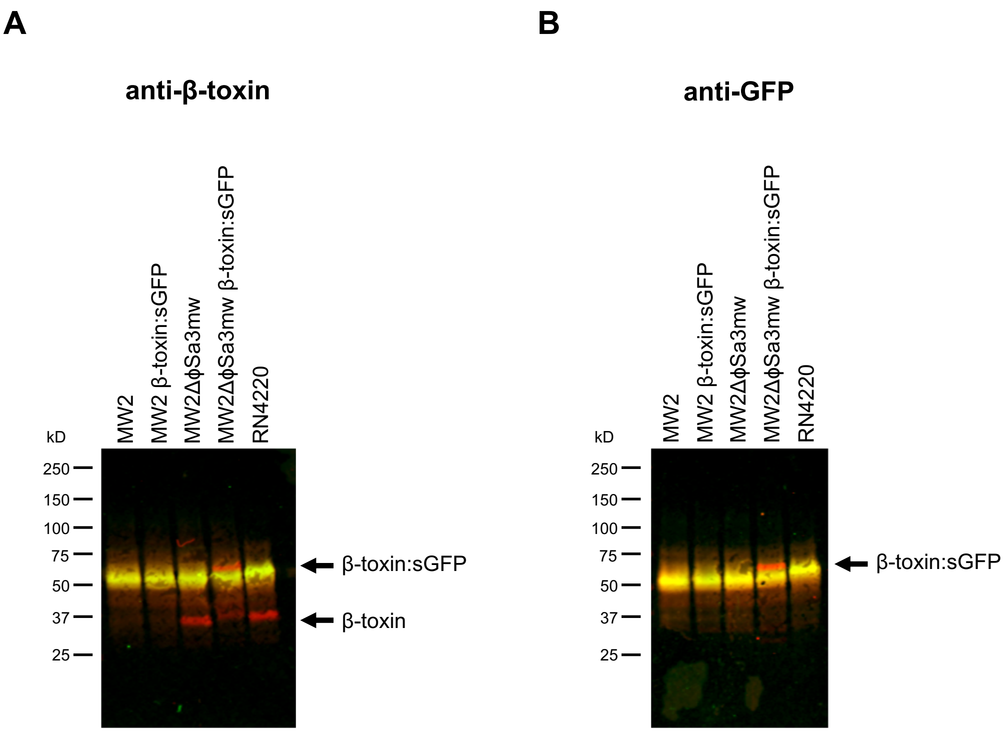

Supplemental Figure 1. Western blot analysis with polyclonal antiserum to β-toxin (**A**) or GFP (**B**) of culture supernatants from *S. aureus.* (**A, B**) Protein A was first quenched with polyclonal antiserum to TSST-1 followed by labeling with goat anti-rabbit IRDye 800CW secondary antibody (**green**, detected in the 800 nm channel). (**A**) β-toxin antiserum was labeled with goat anti-rabbit IRDye 680LT secondary antibody (**red**, detected in the 700 nm channel). Native β-toxin is detected from MW2ΔϕSa3mw and RN4220 at 37 kD while β-toxin:sGFP is detected from MW2ΔϕSa3mw β-toxin:sGFP at 60 kD. β-toxin was not detectable from MW2. (**B**) GFP antiserum was labeled with goat anti-rabbit IRDye 680LT secondary antibody (**red**, detected in the 700 nm channel). β-toxin:sGFP is detected from MW2ΔϕSa3mw β-toxin:sGFP at 60 kD. GFP was not detectable from MW2 supernatant.

Supplemental Figure 2

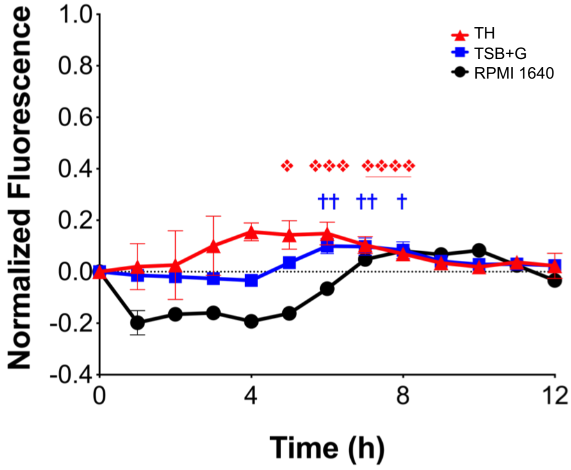

Supplemental Figure 2. Fluorescence signal over time of *S. aureus* MW2 chromosomally expressing β-toxin fused to sGFP in TH (**red triangles**), TSB+G (**blue squares**) or RPMI 1640 (**black circles**). Empty vector control was subtracted from fluorescence readings at corresponding time points to correct for autofluorescence. All results are averages of at least three independent experiments performed in triplicate (mean ± SEM). **Statistical significance** was determined for each time point compared to time zero by one-way ANOVA with Dunnett's multiple comparisons test: TH ❖ $p = 0.029$ (5 h), ❖❖❖ $p = 0.0002$ (6 h), ❖❖❖❖ $p < 0.0001$ (7-8 hrs); TSB+G †† $p = 0.064$ (6 h), †† $p = 0.078$ (7 h), † $p = 0.0342$ (7 h).

Supplemental Figure 3

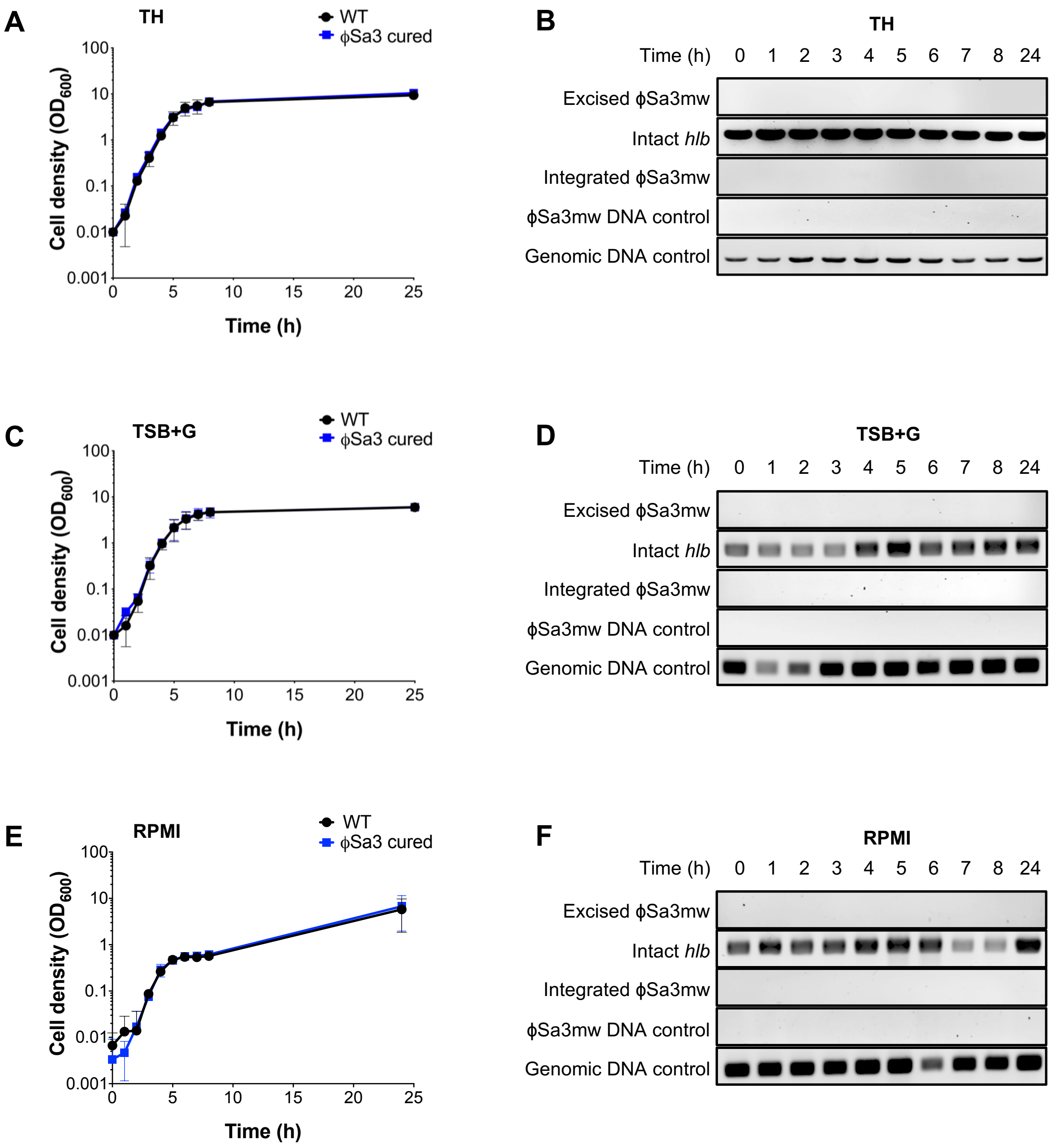

Supplemental Figure 3. (**A, C, E**) Growth curves of MW2 (**black circles**) and MW2ΔϕSa3mw (**blue squares**) grown in (**A**) Todd Hewitt (TH), (**C**) Tryptic Soy Broth supplemented with 2% glucose and 2% NaCl (TSB+G), or (**E**) RPMI 1640 buffered with 10 mM HEPES. (**B, D, F**) PCR analysis of prophage excision in the negative control strain MW2ΔϕSa3mw (phage cured) grown in (**B**) TH, (**D**) TSB+G, or (**F**) RPMI 1640 buffered with 10 mM HEPES. Excised ϕSa3mw: PCR across the *attP* site, present only in excised prophage DNA. Intact *hlb*: PCR across the *attB* site, present only in the absence of integrated prophage. Integrated ϕSa3mw: PCR across the *attR* site, present at the 3'-end of the integration site. ϕSa3mw DNA control: PCR within the ϕSa3mw integrase gene *int*. Genomic DNA control: PCR within the α-toxin gene *hla*.

Supplemental Figure 4

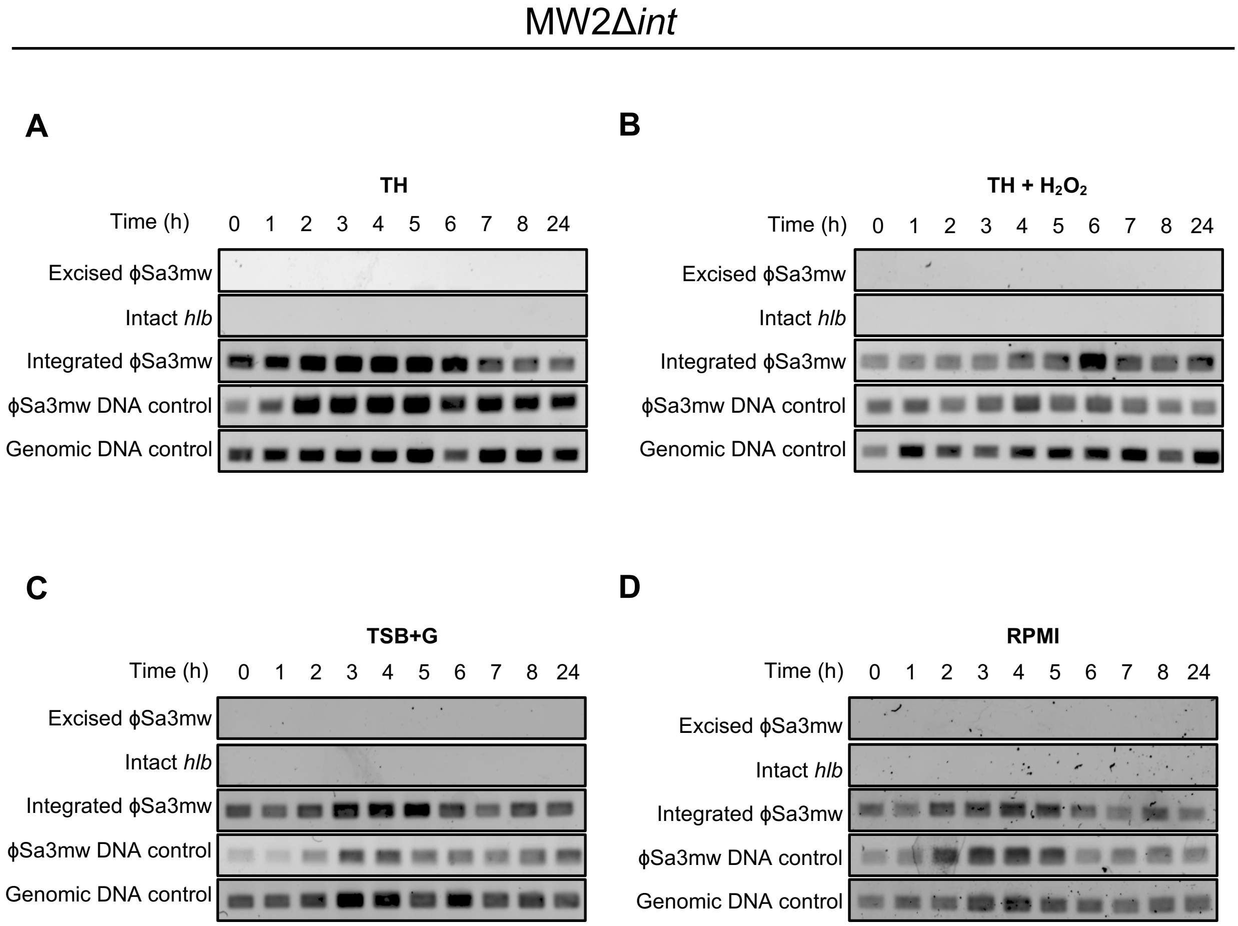

Supplemental Figure 4. PCR analysis of prophage excision in the control strain MW2Δ*int* grown in (**A**) Todd Hewitt (TH), (**B**) TH + 1 mM H_2O_2, (**C**) Tryptic Soy Broth supplemented with 2% glucose and 2% NaCl (TSB+G), or (**D**) RPMI 1640 buffered with 10 mM HEPES. Excised ϕSa3mw: PCR across the *attP* site, present only in excised prophage DNA. Intact *hlb*: PCR across the *attB* site, present only in the absence of integrated prophage. Integrated ϕSa3mw: PCR across the *attR* site, present at the 3'-end of the integration site. ϕSa3mw DNA control: staphylococcal enterotoxin A gene *sea*; genomic DNA control: PCR within the α-toxin gene *hla*.

Table S1. Primers used in this study.

Target	Oligo	Sequence 5’-3’
hlb promoter from MW2	hlb_prom_Forward	CTAGCCCGGGATGATTTATCTAATGGCTTAG
	hlb_prom_Reverse	GTTGGGTACCTATCACTCCTTTTATATAGCTTAC
sgfp from pTH100	sGFP_Forward	GTTAGGTACCATGAGCAAAGGAGAAGAAC
	gfpnew_R	TCGTGAATTCTTATTTGTAGAGCTCATCCATG
Downstream of *hlb*	gBlock DownstreamF	GATGAGCTCTACAAATAGTGCTCAACTAACTAATAACTCGCTTC
	gBlock DownstreamR	CGACTCTAGAGGATCCCCGGAGCGAAAGGAACAATAGGTAGTG
To remove BsaI from pJB38	BsaIdelF	CGGTGAGCGTGGTTCACGCGGTATCA
	BsaIdelR	GCTCCAGATTTATCAGCAATAAACCAG
To amplify gBlock containing sarA-P1-sGFP	pJB38_sarAP1-sGFPF	CGTCTTCAAGAATTCGAGCTGATACGAGACCATATCGCGATTGC
	pJB38_sarAP1-sGFPR	GGATCCCCGGGTACCGAGCTAACATGAGACCATATCGCGAGCT
Upstream of *int*	intKOUpF	TGGTTCGGTCTCGGATAGAATTTTCATTTGGATGTAGAGATT
	intKOUpR	TGGTTCGGTCTCGAAGCGTTCCCTCCTCAAAATTGG
Downstream of *int*	intKODnF	TGGTTCGGTCTCGGCTTTAGGGACCCATTAGGGA
	intKODnR	TGGTTCGGTCTCGAACACTATTTACTATAGGCTTTGATTGGA
	Sa3ExcisF	CGGATCTATAATTAACCTTCAGGCTATCAATAA

Excised, circularized ϕSa3mw (attP)	Sa3ExcisR	CAGTCTAGCTTTGGGGTGTAC
Integrated ϕSa3mw (attR)	Sa3InF	CAAAGAAGCCGGATCTATAATTAAC
	Sa3InR	CGCCACCATCTTCTGC
Intact *hlb* (attB)	hlbscreenF	ATGGTGAAAAAAACAAAATCCAA
	hlbscreenR	CTATTTACTATAGGCTTTGATTGGG
ϕSa3mw *int*	intF	TGCTGGACTAGACAAGTTAAATGAG
	intR	GAGTGTGTCTTAATGCGTGCG
hla	hlaF	CGGTACTACAGATATTGGAAGCA
	hlaR	GATTGCCATATACCGGGTTC
sea	SeaForward.48728039x	GATTCACAAAGGATATTGTTGATAAAT
	SeaReverse.48728032x	GTCCTTGAGCACCAAATAAATC
**qPCR**		
Intact *hlb* (attB)	IntacthlbFqPCR	TAAGAAAGATGATACTGATTTGAA
	IntacthlbRqPCR	TTAATATAAGAAGATTGTCCGATT
Integrated ϕSa3mw (attR)	CJuncFqPCR	CTCCAAACCCAATAAATACTG
	CJuncRqPCR	TTAATATAAGAAGATTGTCCGATT
gyrA	gyrAFqPCR	TGGTTCAATGGATGGAGATGGC
	gyrARqPCR	AGACTGACGGCTCTCTTTCA
